# The Effect of a Maternal Mediterranean Diet in Pregnancy on Insulin Resistance is Moderated by Maternal Negative Affect

**DOI:** 10.3390/nu12020420

**Published:** 2020-02-06

**Authors:** Karen L. Lindsay, Claudia Buss, Pathik D. Wadhwa, Sonja Entringer

**Affiliations:** 1 Departments of Pediatrics, University of California, Irvine, CA 92697, USA; 2 UC Irvine Development, Health and Disease Research Program, University of California, Irvine, CA 92697, USA; claudia.buss@charite.de (C.B.); pwadhwa@uci.edu (P.D.W.); sonja.entringer@charite.de (S.E.); 3 Charité–Universitätsmedizin Berlin, corporate member of Freie Universität Berlin, Humboldt-Universität zu Berlin, and Berlin Institute of Health (BIH), Institute of Medical Psychology, 10117 Berlin, Germany; 4 Departments of Psychiatry & Human Behavior, and Obstetrics & Gynecology, University of California, Irvine, CA 92697, USA

**Keywords:** pregnancy, prenatal diet, Mediterranean diet, negative affect, insulin resistance, homeostasis model assessment of insulin resistance (HOMA-IR)

## Abstract

There is inconsistent evidence that healthy dietary interventions can effectively mitigate the risk of adverse outcomes associated with elevated insulin resistance in pregnancy, suggesting that other moderating factors may be at play. Maternal psychological state is an important factor to consider in this regard, because stress/mood state can directly influence glycemia and a bidirectional relationship may exist between nutrition and psychological state. The objective of this study was to examine the interaction between maternal negative affect and diet quality on third trimester insulin resistance. We conducted a prospective longitudinal study of *N* = 203 women with assessments in early and mid-pregnancy, which included an ecological momentary assessment of maternal psychological state, from which a negative affect score (NAS) was derived, and 24-h dietary recalls, from which the Mediterranean Diet Score (MDS) was computed. The homeostasis model assessment of insulin resistance (HOMA-IR) was computed from third trimester fasting plasma glucose and insulin values. Early pregnancy MDS was inversely associated with the HOMA-IR, but this did not maintain significance after adjusting for covariates. There was a significant effect of the mid-pregnancy MDS*NAS interaction term with the HOMA-IR in the adjusted model, such that a higher negative affect was found to override the beneficial effects of a Mediterranean diet on insulin resistance. These results highlight the need to consider nutrition and affective state concurrently in the context of gestational insulin resistance.

## 1. Introduction

The progressive increase across gestation in maternal insulin resistance is a normal physiological process, with levels peaking in the third trimester, to facilitate shunting of ingested nutrients to the developing fetus [1]. Gestational diabetes mellitus (GDM) is diagnosed when maternal fasting and/or postprandial plasma glucose exceeds normal levels, yet adverse perinatal outcomes have been observed to occur along a spectrum of insulin and glucose levels in pregnancy, even in the absence of overt GDM. Such outcomes include cesarean delivery, preterm birth, macrosomic or large-for-gestational age neonates, shoulder dystocia, neonatal hypoglycemia, and intensive neonatal care [2,3,4]. Furthermore, maternal glucose levels and insulin resistance have been positively and independently associated with early infant weight gain [5], later childhood adiposity [6], and childhood insulin resistance [7]. Thus, effective strategies to control maternal glycemia and moderate insulin resistance are required to promote optimal perinatal and child health outcomes.

Healthy diet and lifestyle is the first line therapy to treat GDM or impaired glucose tolerance in pregnancy [8], yet there is limited evidence from clinical trials showing that dietary interventions are effective at reducing the incidence of adverse clinical outcomes. Although recent studies suggest that adherence to a Mediterranean-style diet may reduce maternal glucose levels, lower GDM risk, and improve glycemic control among women diagnosed with GDM [9,10,11,12], the magnitude of these effects is small, particularly with respect to insulin resistance [11], suggesting that other moderating factors may be at play.

In this regard, maternal psychological state is a critical and potentially modifiable factor to consider in conjunction with prenatal diet/nutritional status for two important reasons. Firstly, maternal psychological state, particularly stress/negative affect (mood), can influence key gestational biological pathways, including glucose-insulin metabolism [13,14]. “Negative affect”, a term to describe low mood state or depressive symptomatology, has been associated with elevated markers of insulin resistance and inflammation in non-pregnant women [15], as well as poor glycemic control in adults with and without type 2 diabetes [16,17]. The mechanisms linking affective state and insulin resistance likely involve dysregulation of immune and endocrine pathways, such as negative affect-induced increases in cytokine production [18] and hypothalamic-pituitary-adrenal (HPA) activity (cortisol) [19,20] that, in turn, may disrupt insulin signaling and sensitivity [21,22]. Secondly, a recursive, bi-directional relationship exists between diet/nutrition and psychological state at both the behavioral and biological levels, yet the biological effects of such interactions have been understudied in the context of pregnancy [23,24]. We have previously reported that pregnant women reporting higher levels of perceived stress consumed foods with higher inflammatory potential and that perceived stress exacerbated the effect of a pro-inflammatory diet on levels of tumor necrosis factor (TNF)-alpha across pregnancy [25]. Furthermore, in non-pregnant women, negative affect has been associated with stress-induced eating [26] and higher frequency of fast-food consumption [27], while consumption of a Mediterranean diet has been associated with lower negative affect [27] and reduced risk of depression [28]. In pregnant cohorts, maternal consumption of a healthy dietary pattern representative of a Mediterranean diet has been associated with lower scores on assessment of postpartum depression risk, while unhealthy dietary patterns have been associated with increased risk [29,30,31]. Therefore, previous studies may have failed to detect effects of prenatal diet on insulin resistance due to a failure to consider the maternal affective state. It is also possible that the effect of diet, affect state, or the interaction between these factors on glycemic outcomes may vary as a function of gestational time point. However, the potential for maternal affect to moderate the effects of diet on gestational glycemia remains to be determined.

Thus, the aim of this study was to investigate whether the interaction effects of maternal negative affect and diet quality measured in early and mid-pregnancy will predict third trimester insulin resistance. We hypothesized that adherence to a Mediterranean diet in pregnancy is associated with lower insulin resistance and that maternal negative affect mitigates this effect. We focused on insulin resistance determined in the third trimester, characterized by the homeostasis model assessment of insulin resistance (HOMA-IR), because insulin resistance is expected to peak in late gestation and to predict the occurrence of adverse perinatal outcomes.

## 2. Materials and Methods

Participants: Healthy pregnant women were recruited for a prospective, longitudinal cohort study on ecological momentary assessments of maternal psychological, behavioral, and biological processes across gestation at the University of California, Irvine (UCI) Development, Health, and Disease Research Program. The UCI Institutional Review Board approved the study, and written informed consent was obtained from all participants.

Women were eligible for inclusion if they were >18 years of age with a singleton, intrauterine pregnancy, and non-diabetic. Women were recruited in the first or early second trimester from obstetric clinics at UCI Medical Center in Orange, California, and affiliated obstetric clinics in surrounding areas. Participants completed up to three assessments at a mean ± standard deviation gestational age of 12.9 ± 1.7, 20.5 ± 1.4, and 30.4 ± 1.4 weeks. Each assessment involved two visits to the laboratory, four days apart. Of a total of *N* = 250 women that were included in the study, those who provided complete data on negative mood assessed with an ecological momentary assessment (EMA) protocol (see below), diet, and the HOMA-IR were included in the present analysis, which amounted to *N* = 184 for the assessment of early-pregnancy diet-mood interaction effects on the HOMA-IR and *N* = 197 for the mid-pregnancy diet-mood interaction effects.

Sociodemographic and clinical data: On the first visit, demographic and clinical data were obtained via structured interviews. Maternal socioeconomic status (SES) was defined as a combination (mean) of maternal educational level (originally assessed in categories from less than high school to advanced degree (master/doctorate) and then recoded into values from 1 to 5) and household income (originally assessed in categories from ≤$15,000 to ≥$100,000 and then recoded into values from 1 to 5). Women were asked about any occurrence of obstetric complications during interviews at each assessment, which was further verified from the medical record at the end of pregnancy. Pregnancies were characterized as “obstetric risk” (dummy variable, “yes” or “no” for presence of obstetric risk during pregnancy), if at least one of the following conditions were confirmed: (a) GDM, (b) preeclampsia or hypertension, (c) anemia, (d) severe infection (e.g., cytomegalovirus, toxoplasmosis, rubella, varicella, mycoplasma, or any sexually transmitted infection), and (e) vaginal bleeding that was present in both middle and late pregnancy (early pregnancy vaginal bleeding was excluded). Pre-pregnancy body mass index (BMI) was computed from self-reported pre-pregnancy weight and measured height (to the nearest 0.1 cm) using a stadiometer, according to the formula Kg/m^2^.

Ecological Momentary Assessment (EMA) protocol to assess negative affect: At the beginning of each prenatal assessment, women were provided with a smartphone programmed with a short questionnaire to assess their emotional and psychological state under ambulatory, naturalistic settings. Each assessment period included two workdays and two weekend days. Diary entries were preprogrammed to signal the participant an average of one time per hour (i.e., every 60 min ± 10 min) during waking hours of the four-day monitoring sequence, reflecting a time-based, stratified, random sampling design. Each EMA diary entry asked the participant to rate on a scale from 0–4 how they currently felt, with respect to the following affective states: (i) anxious, (ii) sad, (iii) angry, and (iv) stressed. Responses were summed to produce a negative affect score (NAS) for each diary entry, with a possible range from 0–4, where higher scores indicated a greater negative affect. The mean NAS score from all diary entries completed over each 4-day assessment period was then computed. The mean NASs from each assessment period were significantly correlated across pregnancy time points (early and mid-pregnancy r = 0.687, *p* < 0.001; mid- and late-pregnancy r = 0.772, *p* < 0.001; early and late pregnancy r = 0.712, *p* < 0.001), but for the present analysis, we considered only the NAS from early and mid-pregnancy as a potential moderator of the association between early and mid-pregnancy Mediterranean diet quality and late-pregnancy HOMA-IR.

Dietary assessments: Dietary intakes were assessed via three interviewer-administered 24-h dietary recalls using the multiple-pass method at the time of each prenatal assessment period. On the first laboratory visit at each assessment, a trained nutritionist conducted the first dietary recall in person with participants, followed by two further dietary recalls by telephone on non-consecutive days, within two weeks of the initial interview. Dietary intake data were entered to the Nutrition Data Software for Research (NDSR) program to generate daily nutrient and food group intake variables, which were averaged over the three dietary recall days at each assessment period. The Mediterranean Diet Score (MDS) was computed as a measure of adherence to the Mediterranean diet, following the method of Chatzi et al. from a US-based pregnant population [32]. The MDS consists of nine components (fruit, vegetables, legumes, wholegrain products, fish, dairy products, red and processed meat, nuts, ratio of monounsaturated to saturated fat), each of which have a defined threshold for optimal daily or weekly intakes (Table S1). For components of the original MDS that had weekly intake thresholds, the equivalent average daily intake threshold was computed by dividing the cut-off value by 7. This was done in order to facilitate dietary data collected by the 24-h recall method, as opposed to food frequency questionnaires. Participants whose average daily intake of a given food component met or exceeded the optimal criteria were assigned a value of 1. If average daily intakes failed to meet the optimal criteria, a value of 0 was assigned. Total points across the MDS components were summed for each participant, generating a composite score ranging from 0 (no dietary adherence) to 9 (maximal dietary adherence). The median dietary intakes and percentage compliance for individual MDS components at each time point of assessment are presented in Table S2. MDS values were significantly correlated across pregnancy time points (early and mid-pregnancy r = 0.181, *p* < 0.014; mid- and late-pregnancy r = 0.269, *p* < 0.001; early and late-pregnancy r = 0.277, *p* < 0.001).

HOMA-IR: Maternal fasting blood collected in the third trimester was assayed for glucose and insulin levels. Antecubital venous blood samples were collected in serum tubes (BD Vacutainer) and samples were allowed to clot for 30 min at room temperature before centrifuging at 4 °C at 1500× *g*. Serum was then separated and stored at −80 °C. Serum glucose was quantitatively determined enzymatically using reagents from Vital Diagnostics (Lincoln, RI). Samples were incubated with the reagent at 37 °C and the absorbance was read at 340/380 nm. Insulin was measured using a radioimmune assay procedure developed by EMD Millipore (St Charles, MO). The homeostasis model assessment of insulin resistance (HOMA-IR) was computed using the formula: (glucose in mg/dL × insulin in µU/mL)/405 [33].

Statistical analyses: Descriptive statistics were used to describe maternal characteristics and the MDS, NAS, and HOMA-IR values across pregnancy. The association between the MDS and NAS within early and mid-pregnancy time points was explored as continuous variables through Pearson correlations and as categorical variables (median split of the NAS, tertiles of the MDS) using the chi-squared test. The HOMA-IR had a non-normal distribution and was log-transformed for subsequent analysis. Maternal age, the SES index, and pre-pregnancy BMI were considered a priori confounding factors due to their established associations with diet quality and insulin resistance. The bivariate associations between additional maternal characteristics (parity, ethnicity, obstetric risk, smoking in pregnancy) and the predictors (mean pregnancy MDS and/or NAS) or outcome (HOMA-IR) variables were explored. Only maternal ethnicity significantly correlated with the HOMA-IR (Spearman rho = −0.246, *p* = 0.001), such that women of Hispanic ethnicity had higher levels. Ethnicity was therefore included as a covariate in regression models alongside the a priori confounding factors.

Linear regression was first used to test the main effects of the MDS and NAS (independent variables) within early and mid-pregnancy time points on late-pregnancy HOMA-IR, with and without adjusting for confounding factors. Each MDS and NAS was mean-centered, and then their product was computed to generate an interaction term for early (T1-MDS*NAS) and mid-pregnancy (T2-MDS*NAS) time points. The linear regression models, as described above, were then repeated to include these interaction terms, as well as the MDSs and NASs within the appropriate time points as independent variables. For ease of graphical interpretation of the interaction effects, MDSs and NASs were categorized such that the difference in HOMA-IR levels was considered across tertiles of the MDS, stratified by higher or lower levels of the NAS (median split). Lastly, since women with a clinical diagnosis of GDM could have skewed the associations with HOMA-IR levels, a sensitivity analysis was performed by repeating the above regression models with GDM cases excluded (*N* = 10). All statistical analyses were performed with SPSS for Macintosh, version 24.0 (SPSS Inc., Chicago, IL, USA), and results were considered statistically significant at the level of *p* < 0.05.

## 3. Results

Descriptive statistics for the study population are presented in Table 1. Mean pre-pregnancy BMI was 26.5 kg/m^2^, and almost half the population was overweight or obese. Median HOMA-IR was 3.12, indicative of a moderately insulin resistant state, as expected in the third trimester. The MDS remained below 2 with a slight increase from early to mid-pregnancy (range 0 to 6), demonstrating poor adherence to the Mediterranean diet in this population (Table 1). Among the MDS components, the poorest compliance was observed for the ratio of monounsaturated to saturated fat, fruits, vegetables, and wholegrains (Table S2). The mean NAS was 0.41 in early pregnancy (range 0.01 to 2.07) and 0.42 in mid-pregnancy (range 0.01 to 3.07), suggesting that, on average, women reported low negative affect across pregnancy (Table 1).

Using unadjusted Pearson correlations, the MDS was not significantly associated with the NAS within time points (T1 MDS-T1 NAS: r = 0.006, *p* = 0.933; T2 MDS-T2 NAS: r = 0.044, *p* = 0.545). Furthermore, Table 2 demonstrates that the distribution of the MDS tertiles across women with higher and lower levels of negative affect, categorized by the population median, were not significantly different either within early pregnancy (Pearson chi-square = 3.331, *p* = 0.189) or mid-pregnancy (Pearson chi-square = 1.095, *p* = 0.578) time points.

In a linear regression model adjusting for the NAS, the MDS was inversely associated with the HOMA-IR only in early pregnancy, although this association did not maintain significance after adjusting for covariates (Table 3). No interaction effect for early pregnancy the MDS*NAS on the HOMA-IR was identified. However, the NAS in mid-pregnancy did moderate the association between the mid-pregnancy MDS and the HOMA-IR, as indicated by the significant association of the T2-MDS*NAS interaction term, which persisted after adjustment for covariates (Table 4).

**Figure 1:**
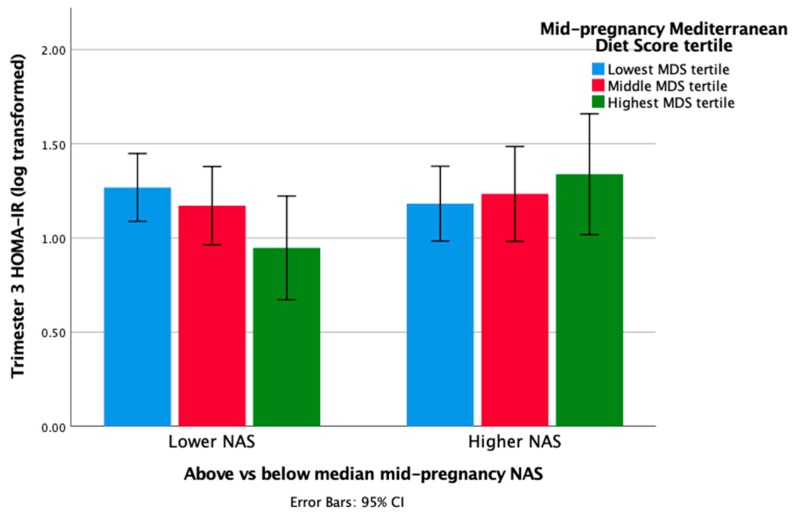
HOMA-IR levels among pregnant women with high and low mid-pregnancy negative affect scores, according to the mid-pregnancy Mediterranean Diet Score tertile.

Figure 1 depicts the nature of this interaction in mid-pregnancy by categorizing the predictor variables, such that a higher negative affect overrides the beneficial effects of a Mediterranean diet on insulin resistance. These results are unchanged after the exclusion of diabetic cases from the analysis (B = 0.148, *p* = 0.041, CI = 0.006, 0.289 for the T1-MDS*NAS interaction term in the adjusted model).

## 4. Discussion

This study presents an important and as-yet-understudied insight into the metabolic effects of the interplay between affective state and diet in pregnancy. Although neither Mediterranean diet adherence nor negative affect were independently associated with the HOMA-IR in late pregnancy, a significant interaction effect was apparent when considering these factors in combination from measures in mid-pregnancy. Specifically, maternal affective state was found to moderate the effects of diet quality on insulin resistance, such that the beneficial influence of a Mediterranean diet on the HOMA-IR measured in the third trimester was only apparent when women were in a state of low negative affect, while Mediterranean diet adherence did not appear to influence the HOMA-IR when women were in a state of high negative affect. However, this effect was specific to mid-pregnancy, since no interaction effect on the third trimester HOMA-IR was detected from the early pregnancy diet and negative affect scores.

In this US-based cohort of pregnant women, adherence to the Mediterranean diet was not found to significantly influence insulin resistance after adjusting for confounders. This finding is somewhat contrary to previous pregnancy studies conducted among European and Middle Eastern populations, in which consumption of a Mediterranean diet was associated with improved glycemic control and reduced risk of GDM [10,11]. The absence of an independent main effect of MDS in our study population on levels of the HOMA-IR may be explained by overall poor adherence to the Mediterranean dietary pattern (total score <2 out of a possible maximum of 9), in particular the low intake of fish and the ratio of monounsaturated to saturated fatty acids, which is reflective of the general poor diet quality in the US. However, the MDS in this study is comparable to that previously reported among the US-based Project Viva pregnancy cohort (mean = 2.7), using the same scoring criteria [32]. However, greater Mediterranean dietary adherence has not consistently been found to be associated with improved glycemic control. In a pregnancy cohort from Spain, adherence assessed in the first trimester was unexpectedly associated with significantly higher fasting blood glucose measured in mid-pregnancy [34]. This suggests that other prenatal moderating factors may be at play, underscoring the need to consider maternal psychological state.

The present study differs from previous studies, examining the effects of a Mediterranean diet in pregnancy by focusing on insulin resistance as the primary outcome measure, rather than glucose control or GDM risk. It is possible that in the short-term, positive effects of a Mediterranean diet on glycemia are manifested through a reduced dietary glycemic load rather than improving insulin sensitivity per se, which may explain the non-significant effects of the intervention diet on serum insulin and the HOMA-IR in the St. Carlos GDM prevention study [11]. While a reduction in maternal fasting glucose will help reduce the incidence of GDM, dietary therapy to control glucose levels has previously been reported to be insufficient to reduce insulin levels among women diagnosed with GDM [35], which may have implications for fetal programming of offspring adiposity and cardiometabolic disease risk [36,37]. Identifying modifiable factors that can effectively control maternal glucose and reduce insulin resistance in pregnancy is therefore warranted; however, focusing on maternal diet alone may be insufficient if the potential moderating effects of the psychological state is not considered.

Maternal negative affect did not correlate with the MDS in the present study, as women reporting higher negative affect states were evenly distributed across the spectrum of Mediterranean diet quality scores in both early and mid-pregnancy. This may be explained by the low score on the monounsaturated-to-saturated fat ratio component, a surrogate marker for olive oil intake in the computation of the MDS [32]. Olive oil is rich in antioxidant compounds, which may reduce oxidative stress and neurotoxicity, which is involved in the pathogenesis of depression [38,39,40]. Although associations between unhealthy dietary patterns and maternal depressive symptoms in the pre- or perinatal period have been previously described [29,41,42,43], no studies have reported on subsequent biological outcomes in the mother or child. Furthermore, we did not detect any main effect of negative affect on insulin resistance, which may be attributed to the generally low NAS values in this “no-risk” sample of women for psychological or psychiatric conditions. As this is the first study, to our knowledge, to investigate the relationship between negative affect and insulin resistance in a pregnancy cohort, it would be of interest to investigate the relationship in a higher risk sample in future studies of pregnant women.

The significant results related to the mid-pregnancy MDS*NAS interaction term in this study reveal that negative affect moderates the effects of a healthy diet on insulin resistance in pregnancy, implying that adherence to a Mediterranean style diet may only be beneficial if negative affect is low. This may have important implications for future research and clinical practice, as simply advising on a healthier dietary intake may not exert a sufficient impact on metabolic control if the maternal mood state and potential depressive symptomatology are not addressed. While many studies suggest that negative affect influences eating behaviors, such as a greater likelihood of binge eating, preference for energy dense foods, and lower restraint intent [44,45], which in turn may lead to insulin resistance over time, we did not find evidence for associations between negative affect and diet quality in this pregnancy cohort. It is possible that the effects of negative affect on insulin resistance operate through maternal biological pathways, which override any potential beneficial effects of a Mediterranean diet. Negative affect may contribute to insulin resistance through dysregulation of the HPA-axis and increased production of inflammatory cytokines. Hyperactivity of the HPA-axis, characterized by elevated salivary cortisol levels, has been reported under momentary conditions of heightened stress and negative affect [46,47], and negative affect has been found to mediate the association between stressful events and cortisol secretion [47,48]. An important role of glucocorticoids is to increase energy substrate availability during times of stress. Cortisol exerts these effects by enhancing muscle protein breakdown and promoting adipose tissue lipolysis and hepatic gluconeogenesis, contributing to elevated circulating glucose concentrations [49]. Furthermore, glucocorticoid receptors are more abundant on visceral than subcutaneous adipose tissue, and thus, under conditions of subclinical hypercortisolism, visceral fat accumulation occurs through differentiation and proliferation of adipocytes and fat redistribution from peripheral to central depots, promoting hepatic insulin resistance [50]. Experimental and observational studies in non-pregnant adults have indeed demonstrated that elevated evening cortisol levels and a heightened cortisol response to endocrine stress is associated with insulin resistance and type 2 diabetes [20,51,52]. With respect to inflammation, negative affect and a depressed mood can directly provoke proinflammatory cytokine production [53,54] and prime the inflammatory response by inducing a larger cytokine increase, following a stressful situation or antigen challenge [55]. Elevated proinflammatory cytokines are involved in the pathogenesis of insulin resistance. For example, TNF-alpha induces phosphorylation of the insulin receptor substrate 1 on serine residues via activation of cellular stress response kinases, leading to insulin signaling impairment [56,57,58]. A low-grade inflammatory state has also been shown to induce adipose tissue insulin resistance in late pregnancy [59].

Although these mechanistic pathways, by which negative affect promotes insulin resistance, were not tested in the current study, our findings suggest that the adverse effects of negative affect override any potential protective effects of a Mediterranean diet on insulin sensitivity. A similar interaction effect between diet and psychological state was demonstrated by Kiecolt-Glaser et al., in an experimental study among non-pregnant women [60], in which the presence of prior day stressors exacerbated the postprandial inflammatory response to a healthy Mediterranean-type meal, causing the response to appear similar to that seen with a high saturated fat meal. Our results are somewhat contrary to expectations that the highest insulin resistant state would be observed among women with the combination of worst diet quality and high negative affect. However, only the usual dietary intakes during pregnancy are captured in this study, which are not necessarily reflective of longer-term pre-pregnancy dietary habits. It is possible that women consciously make healthier dietary choices during pregnancy compared to their typical pre-pregnancy diet, and this may occur regardless of current mood state. However, if such women typically had unhealthy dietary intakes over the longer-term, it is unlikely that a short-term period of prenatal dietary improvement could undo the detrimental effects on insulin resistance, particularly against a background of normal gestational increases in insulin resistance. Furthermore, we did not detect any interaction effect of early pregnancy measures of negative affect and Mediterranean dietary adherence on the HOMA-IR, which may be explained by the longer duration between the exposure and outcome measurement time points. The MDS was also slightly lower in early compared to mid-pregnancy, possibly attributed to altered appetite and nausea that is typically experienced in the first trimester, which may have impacted any potential beneficial effect of diet on insulin resistance, even in those with the lower negative affect.

Strengths of this study include the diverse population with respect to ethnicity, age, pre-pregnancy BMI, and SES, as well as the longitudinal collection of maternal diet and affect across pregnancy, facilitating analysis of sensitive time points in gestation, during which diet and affect state may differentially influence insulin resistance. Furthermore, the use of EMA technology to record psychological states in real-time naturalistic settings significantly reduces the risk of recall and saliency bias compared to commonly used retrospective methods. However, interpretation of the results of this study may be limited by the low variation in maternal negative affect. Additionally, as is the case with all non-laboratory-based dietary assessment studies, an unavoidable risk of respondent and recall bias in reporting of dietary intakes exists, although the use of multiple 24-h recalls reduces this risk compared to more widely used food frequency questionnaires. Lastly, we acknowledge the possibility for unmeasured confounding in our analysis from underlying medical conditions, such as non-alcoholic fatty liver disease, which may influence insulin resistance beyond diet or affect state.

## 5. Conclusions

This study provides evidence that the effects of a Mediterranean diet on gestational insulin resistance may be moderated by maternal affect state. This may hold particular relevance in populations such as the US, where the average population diet quality falls far below that of other populations with closer origins to the Mediterranean diet and in which the prevalence of negative affect states and depression is high. Concurrently addressing maternal diet quality and psychological state in pregnancy could help improve the maternal metabolic profile, which may hold implications for long-term maternal health outcomes, as well as fetal programming of adiposity and cardiometabolic disease risk.

## Figures and Tables

**Table 1:** Maternal sociodemographics, insulin resistance, the Mediterranean Diet Score (MDS) and negative affect score (NAS) (*N* = 203).

Maternal Characteristic	Value
Age (years; mean ± SD)	27.95 ± 5.30
SES Index (mean ± SD)	3.23 ± 0.93
Pre-pregnancy BMI (kg/m^2^; mean ± SD)	26.50 ± 6.46
BMI category (N (%))	
<25.0 kg/m^2^	105 (51.7)
25.0–29.9 kg/m^2^	51 (25.1)
>29.9 kg/m^2^	43 (21.2)
Ethnicity (N (%))	
Hispanic	87 (42.9)
Non-Hispanic	107 (52.7)
Primiparous (N (%))	84 (41.4)
Smoking during pregnancy: yes (N (%))	15 (7.4)
Obstetric risk condition: yes (N (%))	44 (21.7)
T3 HOMA-IR (median (IQR))	3.12 (2.63)
T1 MDS (mean ± SD)	1.86 ± 1.28
T2 MDS (mean ± SD)	1.98 ± 1.34
T1 NAS (mean ± SD)	0.41 ± 0.38
T2 NAS (mean ± SD)	0.42 ± 0.43

Body mass index (BMI); homeostasis model assessment of insulin resistance (HOMA-IR); interquartile range (IQR); Mediterranean Diet Score (MDS); negative affect score (NAS); socioeconomic status (SES); early-pregnancy (T1); mid-pregnancy (T2); late pregnancy (T3).

**Table 2:** Distribution of women in each tertile of the MDS according to the median split of the NAS within early and mid-pregnancy time points.

	Early Pregnancy		Mid-Pregnancy	
	Below Median NAS	Above Median NAS	Below Median NAS	Above Median NAS
Lowest MDS tertile	43 (48.9%)	33 (37.9%)	42 (43.8%)	36 (37.9%)
Middle MDS tertile	20 (22.7%)	30 (34.5%)	28 (29.2%)	27 (28.4%)
Highest MDS tertile	25 (28.4%)	24 (27.6%)	26 (27.1%)	32 (33.7%)

Mediterranean Diet Score (MDS); negative affect score (NAS).

**Table 3:** Main effect of the maternal MDS and NAS in early and mid-pregnancy on the third trimester HOMA-IR.

**Unadjusted Analysis**	**B**	**Std. Error**	**95% CI**		***p*-Value**
T1 MDS	−0.101	0.039	−0.179	−0.024	0.011
T1 NAS	0.139	0.139	−0.135	0.413	0.318
**Adjusted Analysis**					
T1 MDS	−0.059	0.037	−0.133	0.015	0.118
T1 NAS	0.147	0.130	−0.110	0.404	0.262
Maternal age	−0.027	0.009	−0.045	−0.009	0.004
SES index	0.012	0.062	−0.111	0.135	0.845
Pre-pregnancy BMI	0.029	0.007	0.014	0.043	<0.001
Ethnicity	−0.190	0.110	−0.407	0.028	0.087
**Unadjusted Analysis**	**B**	**Std. Error**	**95% CI**		***p*-Value**
T2 MDS	−0.038	0.035	−0.108	−0.031	0.277
T2 NAS	0.050	0.111	−0.168	0.269	0.650
**Adjusted Analysis**					
T2 MDS	0.006	0.034	−0.062	0.073	0.867
T2 NAS	0.095	0.104	−0.110	0.300	0.363
Maternal age	−0.024	0.009	−0.042	−0.006	0.008
SES index	−0.001	0.059	−0.117	0.114	0.986
Pre-pregnancy BMI	0.030	0.007	0.016	0.044	<0.001
Ethnicity	−0.208	0.100	−0.406	−0.001	0.040

Body mass index (BMI); Mediterranean Diet Score (MDS); negative affect score (NAS); socioeconomic status (SES); early-pregnancy (T1); mid-pregnancy (T2).

**Table 4:** Interaction effects of the maternal MDS*NAS in early and mid-pregnancy on the third trimester HOMA-IR.

**Unadjusted Analysis**	**B**	**Std. Error**	**95% CI**		***p*-Value**
Interaction T1-MDS*NAS	0.007	0.125	−0.239	0.253	0.957
**Adjusted Analysis**					
Interaction T1-MDS*NAS	−0.043	0.117	−0.274	0.188	0.711
Maternal age	−0.027	0.009	−0.045	−0.009	0.004
SES index	0.011	0.062	−0.112	0.135	0.859
Pre-pregnancy BMI	0.029	0.007	0.014	0.043	<0.001
Ethnicity	−0.187	0.111	−0.406	0.032	0.093
**Unadjusted Analysis**	**B**	**Std. Error**	**95% CI**		***p*-Value**
Interaction T2-MDS*NAS	0.173	0.071	0.033	0.312	0.035
**Adjusted Analysis**					
Interaction T2-MDS*NAS	0.142	0.067	0.010	0.274	0.035
Maternal age	−0.025	0.009	−0.043	−0.008	0.005
SES index	−0.005	0.058	−0.120	0.109	0.927
Pre-pregnancy BMI	0.027	0.007	0.013	0.041	<0.001
Ethnicity	−0.199	0.100	−0.395	−0.002	0.047

Body mass index (BMI); Mediterranean Diet Score (MDS); negative affect score (NAS); socioeconomic status (SES); early-pregnancy (T1); mid-pregnancy (T2).

## Supplementary Materials

The following are available online at https://www.mdpi.com/2072-6643/12/2/420/s1: Table S1: Description of food groups and computation of the MDS, Table S2: Dietary intakes and population compliance to individual MDS components by stage of pregnancy.
