# Supplementary material for: The Effect of a Maternal Mediterranean Diet in Pregnancy on Insulin Resistance is Moderated by Maternal Negative Affect
Source: Nutrients. 2020 Feb 6;12(2):420. doi: 10.3390/nu12020420 (PMC7071160; doi:10.3390/nu12020420)
Supplement: Supplementary file 1 [file nutrients-12-00420-s001.zip › Supplementary tables/Supplementary Table S1.docx]

| Table S1: Description of food groups and computation of the Mediterranean Diet Score* for pregnant women | | | |
| --- | --- | --- | --- |
| **Food groups** | **Description** | **Cut-off** | **Adapted for current cohort** |
| Fruits | All fruits and 100% fruit juices | ≥ 3 servings/day | ≥ 3 servings/day |
| Vegetables | All raw and cooked vegetables | ≥ 3 servings/day | ≥ 3 servings/day |
| Legumes | Dried beans, lentils, peas, soups (split pea), tofu, soymilk | ≥ 1.5 servings/week | ≥ 0.214/day |
| Whole grain products | Whole wheat bread and flour, whole wheat pasta, brown rice, rusks, whole grain breakfast cereals, couscous, semolina | ≥ 3 servings/day | ≥ 3 servings/day |
| Fish & shellfish | Fresh-water and sea-water fish; preserved fish such as salted fish, canned fish; shellfish (squid, prawns, mollusks) | ≥ 2 servings/week | ≥ 0.286/day |
| Dairy products | Milk, yogurt, cheese, custard, milk puddings, other milk products | ≥ 2 servings/day | ≥ 2 servings/day |
| Red and processed meat | Beef, pork, lamb, goat, veal, sausages, bacon, salami, ham | < 4.5 servings/week | < 0.643/day |
| Nuts & seeds | Peanuts, almonds, sunflower seeds, cashews, walnuts | ≥ 2 servings/week | ≥ 0.286/day |
| Ratio of monounsaturated to saturated lipids | Monounsaturated:saturated lipids/day | ≥ 1.6 | ≥ 1.6 |
| *using methods as described by Chatzi et al. [29] and modified for dietary assessment by 24-hour recalls in the present study cohort | | | |
