# Supplementary material for: The Effect of a Maternal Mediterranean Diet in Pregnancy on Insulin Resistance is Moderated by Maternal Negative Affect
Source: Nutrients. 2020 Feb 6;12(2):420. doi: 10.3390/nu12020420 (PMC7071160; doi:10.3390/nu12020420)
Supplement: Supplementary file 1 [file nutrients-12-00420-s001.zip › Supplementary tables/Supplementary Table S2.docx]

| **Table S2:** Dietary intakes and population compliance to individual MDS components in early and mid-pregnancy | | | | |
| --- | --- | --- | --- | --- |
|  | **Early pregnancy** | | **Mid pregnancy** | |
| **Food group** | **Median (IQR) intake** | **% compliance** | **Median (IQR) intake** | **% compliance** |
| Fruits | 1.08 (1.65) | 10.9 | 1.05 (1.80) | 14.0 |
| Vegetables | 1.71 (1.49) | 17.6 | 1.86 (1.74) | 19.5 |
| Legumes | 0.02 (0.33) | 33.9 | 0.06 (0.33) | 35.3 |
| Whole grain products | 1.44 (1.83) | 16.3 | 1.59 (1.71) | 16.3 |
| Fish & shellfish | 0 (0) | 14.5 | 0 (0.23) | 23.5 |
| Dairy products | 1.60 (1.23) | 32.1 | 1.63 (1.14) | 34.8 |
| Red and processed meat | 1.65 (1.92) | 21.3 | 1.64 (2.08) | 17.6 |
| Nuts & seeds | 0 (0.62) | 34.4 | 0 (0.50) | 34.8 |
| Ratio monounsaturated: saturated fats | 1.01 (0.27) | 2.7 | 1.03 (0.28) | 5.0 |
| Mean (SD) total MDS |  | 1.84 (1.26) |  | 2.01 (1.36) |
| MDS, Mediterranean diet score; IQR, interquartile range; SD, standard deviation. | | | | |
